# Myocardial Computed Tomography Perfusion: One More Piece on The Board

**DOI:** 10.36660/abc.20190671

**Published:** 2019-12

**Authors:** Gabriel Blacher Grossman

**Affiliations:** 1 Serviço de Medicina Nuclear do Hospital Moinhos de Vento, Porto Alegre, RS - Brazil; 2 Clínica Cardionuclear - Instituto de Cardiologia, Porto Alegre, RS - Brazil

**Keywords:** Coronary Artery Disease/physiopathology, Myocardial Ischemia, Tomography, Emission Computed/methods, Cineangiography/methods, Myocardial Perfusion, Imaging, Cardíac Catheterization, Exercise.

The most appropriate way to evaluate patients with stable coronary artery disease (CAD) and the subsequent definition of the therapeutic approach has been the subject of debate in recent years. For several years the anatomical evaluation was considered sufficient to indicate myocardial revascularization. The emergence of several methods of non-invasive functional evaluation in clinical practice as well as data from observational studies demonstrating that there is a level of ischemia above which a revascularization strategy might result in benefit regarding cardiovascular events raised doubts whether an strategy based in coronary anatomic findings was the best option.^1,2^ This questioning changed the paradigm of CAD evaluation. Although randomized clinical trials have failed to demonstrate that the extent of ischemia can determine which patients would benefit from a revascularization strategy,^3-5^ the fact that the presence of moderate to severe ischemia is undeniably a marker of cardiovascular risk has lead functional evaluation to become a fundamental part in the management of patients with stable CAD.

In this context, Ker et al.^6^ in this edition of the *Arquivos Brasileiros de Cardiologia* evaluated 35 patients undergoing a simultaneous pharmacologic stress protocol of myocardial perfusion evaluation by computed tomography angiography (angio-CT) and single-photon emission computed tomography (SPECT) and compared the sensitivity of the methods using the presence of obstructive lesion evidenced by angio-CT greater than 50% as the gold standard for the presence of significant CAD.^6^

For the detection of obstructive CAD, the evaluation of myocardial perfusion by angio-CT had an area under the curve of 0.84 [confidence interval of 95% (95%CI): 0.67 to 0.94, P < 0.001]. SPECT had an area under the curve of 0.58 (95%CI: 0.40 to 0.74, P < 0.001). The sensitivity of SPECT to detect stenosis greater than 50% determined by angio-CT was 66%, with specificity of 50%. The sensitivity of perfusion angio-CT for detection of obstructive CAD was 93%, with specificity of 75% for the detection of absence of obstructive CAD by coronary angiography. In this study, false positives were considered when ischemia was present in a SPECT study with absence of obstructive CAD demonstrated by angio-CT. The authors concluded that the evaluation of myocardial perfusion by angio-CT presents satisfactory results in comparison with SPECT and that angio-CT can exclude false-positives of SPECT studies.

Although it is noteworthy the importance of developing new techniques to improve the evaluation of patients with CAD, it is fundamental to analyze which gold standard is used to test the accuracy of new diagnostic modalities. It is recognized that one of the limitations of the CT angiography is a specificity and positive predictive values suboptimal and a tendency to overestimate coronary lesions, being its sensitivity and negative predictive value excellent, providing necessary reassurance to exclude significant CAD,^7^ This limitation hinders a more adequate analysis of the diagnostic accuracy of the methods in this study, because the method used as a reference has its main limitation in predicting the presence of ischemia. In addition, anatomy evaluated by CT served as the gold standard for assessing the sensitivity and specificity of CT perfusion, that is, the tested method served as its own gold standard. In the CORE 320 study, cardiac catheterization was used as a reference for the diagnosis of CAD.^8^ In the CORE 320 study, the sensitivity of the angio-CT was 88% and the specificity was 55%, and SPECT presented sensitivity and specificity of 62% and 67%, respectively. Recently, catheterization associated with the measurement of fractional flow reserve (FFR) has been considered the method of choice for testing the diagnostic accuracy of other functional methods.

On the other hand, the presence of perfusion abnormalities in a functional test in the absence of obstructive CAD cannot always be categorized as "false-positive" results. It is increasingly recognized the role of coronary microcirculation dysfunction as a cause of ischemia and symptoms, generating the term microvascular angina.^9^ In this sense, methods that quantify the absolute coronary flow, such as positron emission tomography (PET), allow the quantification of myocardial flow and coronary flow reserve and can detect microvascular dysfunction. Unfortunately, cardiac PET is not a reality in Brazil.

Several publications in the literature do not demonstrate similar sensitivity and specificity of SPECT in comparison of what was determined by Ker et al.^6^ In a meta-analysis comparing SPECT, magnetic resonance and PET, using coronary catheterization without FFR as a gold standard, Jaarsma et al.^10^ reported a sensitivity of 84% and specificity of 61% for SPECT.^10^ In a recent meta-analysis that used coronary catheterization with FFR as a gold standard, the sensitivity and specificity of SPECT was 74% and 79%, respectively.^11^

SPECT is an excellent non-invasive method for evaluating stable CAD, predominantly in patients with intermediate risk, or even in high-risk patients to helP in the planning of the therapeutic approach. In addition, it is possible to use exercise as the stress protocol in patients who have the adequate functional capacity and good clinical condition. It is well known that an exercise stress protocol is the method of choice to evaluate patients with suspected or established CAD.

In conclusion, to assess the accuracy of a diagnostic method, it is critical to choose the right gold standard. The use of anatomic criteria based on the angio-CT findings does not invalidate the study of Ker et al.,^6^ which opens a perspective for a new non-invasive technique that can assist in the proper management of patients with stable CAD, as well as creates the perspective of new research in this area. In the future, myocardial perfusion by angio-TC may be aggregated to the existing diagnostic armamentarium for the evaluation of patients with stable CAD, always taking into account the characteristic of the patient, and especially the functional capacity and possibility of exercise. In this context, non-invasive diagnostic methods that allow to perform exercise stress protocols should be the first choice.
